# The diagnostic challenge of coexistent sarcoidosis and thyroid cancer – a retrospective study

**DOI:** 10.1186/s12885-020-07745-w

**Published:** 2021-02-07

**Authors:** Vera Wenter, Nathalie L. Albert, Freba Ahmaddy, Marcus Unterrainer, Julia Hornung, Harun Ilhan, Peter Bartenstein, Christine Spitzweg, Nikolaus Kneidinger, Andrei Todica

**Affiliations:** 1 Department of Nuclear Medicine, University Hospital, LMU Munich, Marchioninistr. 15, 81377 Munich, Germany; 2 Department of Radiology, University Hospital, LMU Munich, Munich, Germany; 3 Comprehensive Cancer Center (CCC LMU) and Interdisciplinary Center for Thyroid Carcinoma (ISKUM), University Hospital, LMU Munich, Munich, Germany; 4 Department of Internal Medicine IV, University Hospital, LMU Munich, Munich, Germany; 5 Department of Internal Medicine V, University Hospital, LMU Munich, Comprehensive Pneumology Center (CPC-M), Helmholtz Zentrum München, Member of the German Centre for Lung Research (DZL), Munich, Germany

**Keywords:** Thyroid cancer, Thyroid gland, Sarcoidosis, Radioiodine therapy

## Abstract

**Background:**

Sarcoid lesions may mimic metastatic disease or recurrence in thyroid cancer (TC) patients as both diseases may affect the lungs and lymph nodes. We present the first study to systematically evaluate the clinical course of patients with (TC) after adjuvant radioactive iodine therapy (RIT) and concomitant sarcoidosis of the lung or the lymph nodes.

**Methods:**

We screened 3285 patients and retrospectively identified 16 patients with TC (11 papillary thyroid cancer (PTC), 3 follicular thyroid cancer (FTC), 1 oncocytic PTC, 1 oncocytic FTC) and coexisting sarcoidosis of the lung and/or the lymph nodes treated at our institute. All patients had undergone thyroidectomy and initial adjuvant RIT. Challenges in diagnosing and the management of these patients were evaluated during long term follow-up (median 4.9 years (0.8–15.0 years)).

**Results:**

Median age at first diagnosis of TC was 50.1 years (33.0–71.5 years) and of sarcoidosis 39.4 years (18.0–63.9 years). During follow-up, physicians were able to differentiate between SA and persistent or recurrent TC in 10 of 16 patients (63%). Diagnosis was complicated by initial negative thyroglobulin (Tg), positive Tg antibodies and non-specific imaging findings. Histopathology can reliably distinguish between SA and TC in patients with one suspicious lesion.

**Conclusion:**

Physicians should be aware of the rare coexistence of sarcoidosis and TC. Lymphadenopathy and pulmonary lesions could be metastases, sarcoidosis or even a mix of both. Therefore, this rare patient group should receive a thorough work up including histopathological clarification and, if necessary, separately for each lesion.

## Background

Sarcoidosis is an idiopathic inflammatory disease that is characterized by granuloma formation in affected organs, mainly the lungs and the lymphatic systems of the body [1]. However, the clinical course of sarcoidosis can be highly variable and multiple organ systems may be involved [1, 2]. Although the skin, eyes, bones, liver, spleen, heart, upper respiratory tract and nervous system can be affected, as stated above sarcoidosis primarily involves the lungs and thoracic lymph nodes [3]. Papillary and follicular thyroid cancer are the most common differentiated thyroid cancers (TC) [4, 5]. While papillary thyroid cancer (PTC) metastasizes predominantly in locoregional lymph nodes, follicular thyroid cancer (FTC) tends to invade blood vessels and metastasizes by haematogenous spread to distant sites, most commonly lungs and bones. Thus, the manifestation of both TC and sarcoidosis can be similar, especially when the lung and lymph nodes are affected. Occasionally, TC and sarcoidosis can coexist and manifestation of sarcoidosis can mimic metastatic TC which is up to now only described in individual case reports [6–17]. In a certain percentage of cases the clinical course of sarcoidosis is completely asymptomatic and silent [1]. Likewise, metastatic TC can be clinically unapparent. Thus, diagnosing and the management of concomitant TC and sarcoidosis can be challenging and need special attention. Therefore, we present the first study which systematically evaluates the clinical course of patients with TC after adjuvant radioactive iodine therapy (RIT) and concomitant sarcoidosis.

## Methods

We retrospectively reviewed patients with TC who were followed up at our institution between 1996 and 2018. Patients with sarcoidosis of the lung or lymph nodes diagnosed before or after initial diagnosis of TC were included. For our study 3285 patients were screened and the final study population consisted of 16 patients. TNM staging was based on the AJCC 8th edition in all patients. All patients underwent total thyroidectomy with (*N* = 10) or without (*N* = 6) lymphadenectomy (LAE). RIT was performed either after thyroid hormone withdrawal under hypothyroidism (TSH ≥30 μU/ml) or after administration of 0.9 mg recombinant human thyrotropin alfa (rhTSH; Thyrogen®, Sanofi-Aventis, Frankfurt, Germany) on two consecutive days with administration of radioiodine ^131^I 24 h after the second injection. Planar whole-body scans (WBS) were obtained 72 h after oral administration of ^131^I using either the Siemens Symbia T, the Symbia Intevo or the e.cam (all Siemens Healthcare GmbH, 91,052 Erlangen, Germany) dual-head multifunctional gamma camera with high-energy high-resolution all-purpose collimator. An additional SPECT/low-dose-CT of the neck and thorax were performed to increase the diagnostic accuracy (*N* = 10/16). During the 1st year, follow-up examinations of TC, including physical examination, ultrasound of the neck and laboratory examination, were performed every 3 months. Additionally, 6 to 9 months after the 1st RIT a diagnostic ^131^I WBS (370 MBq/10 mCi) was performed in hypothyroidism or after rhTSH. An additional SPECT/(low-dose-CT) was performed if local or distant uptake was present in the planar WBS. If TC did not persist or recur, follow-up examinations were extended to a six-month interval in the 2nd year and annually thereafter. In case of suspected tumour recurrence additional radiological examinations, such as X-ray of the chest, CT thorax and MRI of the neck, or whole-body 2-deoxy-2-^18^Ffluoro-D-glucose (^18^F FDG)-PET/(CT) scans were performed. For the diagnosis and treatment of sarcoidosis patients were referred to the department of pneumology. The diagnosis of sarcoidosis was established through clinical, laboratory, radiologic investigations, and / or bronchoalveolar lavage and histopathology. Once the diagnosis was established further work-up was performed including pulmonary function tests, peripheral blood counts, serum chemistry, urine analysis; and tuberculin skin test if indicated [1]. To assess psycho-oncological distress during follow-up the German version of the National Comprehensive Cancer Network (NCCN) Distress-thermometer was used which consists of a scale from 0 to 10 [18].

### Endpoints

The aim of our study was the assessment of the clinical course of patients with TC after adjuvant RIT and concomitant sarcoidosis. Furthermore, we evaluated the diagnostic difficulties in differentiating between TC and sarcoidosis and the patient management.

## Results

### Patient characteristics

An overview on patient characteristics is reported in Table 1. Overall, 16 patients with TC who were treated at our centre between 1996 and 2018 and were additionally diagnosed with sarcoidosis were retrospectively identified. Eleven out of 16 patients had papillary thyroid cancer (PTC), three had follicular thyroid cancer (FTC), one had oncocytic PTC, and one oncocytic FTC. The study group consisted of nine female and seven male patients. Median age at diagnosis of TC was 50.1 years (33.0–71.5 years) and of sarcoidosis 39.4 years (18.0–63.9 years). Median follow-up time was 4.9 years (0.8–15.0 years).

**Table 1:** Patients’ baseline characteristics

**Age at diagnosis of TC (in years)**	50.1 years (33.0–71.5 years)
**age at diagnosis of sarcoidosis (in years)**	39.4 years (18.0–63.9 years)
**gender**	female *N* = 9 male *N* = 7
**initial radio-activity (MBq)**	3687 MBq, 100 mCi (2002–9200 MBq, 54–249 mCi)
**cumulative radio-activity (MBq)**	6658 MBq, 180 mCi (2067–35,510 MBq, 56–960 mCi)
**Initial stimulated Tg**	< 0.5 ng/ml *N* = 2 increased *N* = 14
**Tg recovery**	normal *N* = 15 disturbed N = 1
**Tg antibodies**	positive *N* = 4 negative *N* = 5 missing *N* = 7
**histology**	PTC *N* = 11 FTC *N* = 3 OPTC *N* = 1 OFTC *N* = 1
**tumor stage**	pT1 *N* = 10 pT2 *N* = 4 pT3 *N* = 1 pT4 *N* = 1
	pN0 *N* = 4 pN1 *N* = 6 pNx *N* = 6
	pR0 *N* = 11 pR1 *N* = 1 pRx *N* = 4
	cMo *N* = 15 cM1 (lung) *N* = 1

*PTC* papillary thyroid carcinoma, *FTC* follicular thyroid carcinoma, *OFTC* oncocytic follicular thyroid carcinoma, *OPTC* oncocytic papillary thyroid carcinoma, *p* histopathologic, *c* clinical, *T* tumour, *N* lymph nodes, *M* metastasis, *R* resection-boundaries, *Tg* thyroglobulin

First adjuvant RIT was performed 1.5 months (0.4–5.8 months) after thyroidectomy either after endogenous (*N* = 11) or exogenous stimulation (*N* = 5). Median ^131^I dose was 3687 MBq (2002–9200 MBq, 54–249 mCi). At the time of 1st RIT two patients presented with negative Tg (one patient with normal and one with disturbed Tg recovery). Tg antibodies were positive in four patients, negative in five patients, and not available in seven patients due to the retrospective study design.

### Differentiation between sarcoidosis and thyroid cancer

**Fig. 1:**
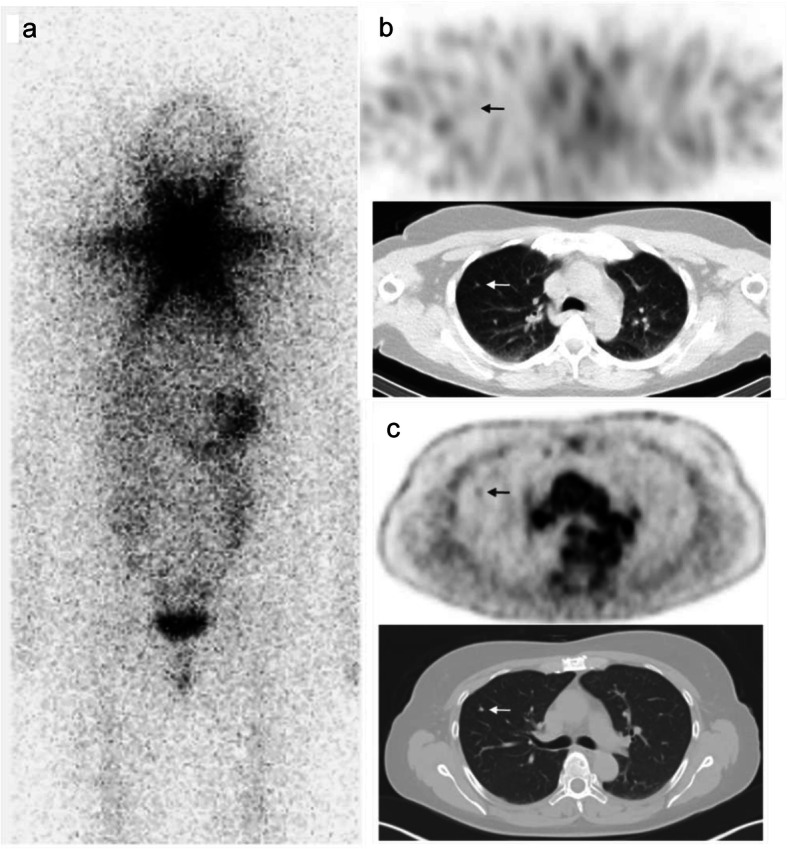
**a**-**c** patient with iodine-negative pulmonary lesions. **a** shows the posttherapeutic ^131^I WBS of patient #8 after 1st RIT with significant uptake at the thyroid bed. Iodine-negative pulmonary lesions were diagnosed in the ^131^I SPECT/CT after 1st RIT (**b**). Consequently, the patient was mistakenly upgraded cM1 and treated with an adjuvant second RIT and with TSH-suppression. Second RIT showed an iodine-positive cervical lymph node (cN1), but still iodine-negative lung nodules (2nd ^131^I WBS not shown). ^18^F FDG PET/CT (**c**) revealed ^18^F FDG positive progressive pulmonary lesions which were still falsely considered as pulmonary metastases during follow-up despite negative Tg. After 1.5 years sarcoidosis was proven histopathologically by thoracoscopic atypical lung resection during follow-up. After diagnosing sarcoidosis the patient was downgraded to cM0. The patient remained disease-free during follow-up

**Fig. 2:**
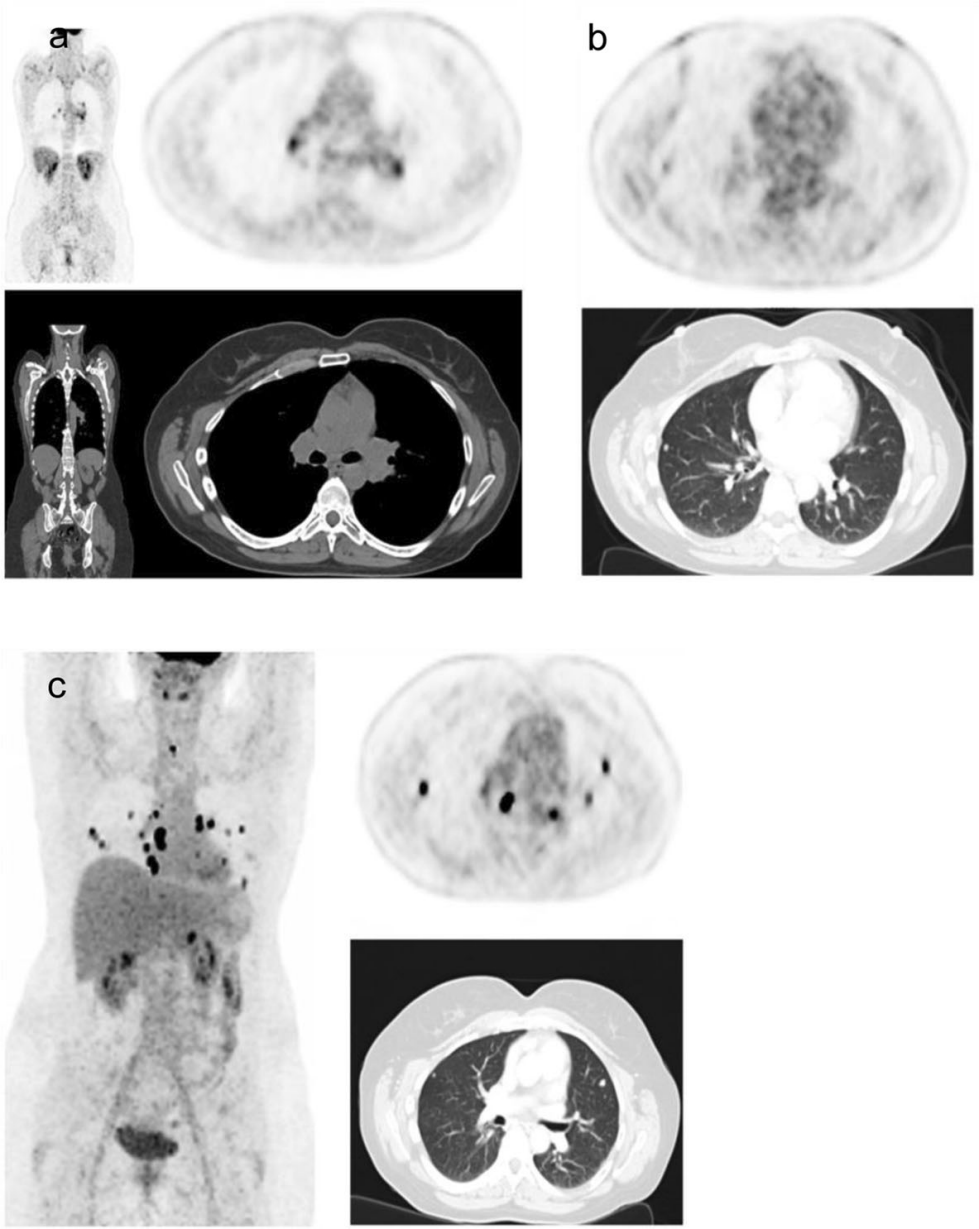
**a**-**c** Patient with ^18^F FDG positive sarcoidosis and TC: Patient #10 presented with hilar lymphadenopathy in the ^18^F FDG PET/CT (SUVmax 8.3; **a**) and elevated Tg of 9.0 ng/ml one year after RIT. Histopathologically and clinically acute sarcoidosis was confirmed. The elevated Tg remained morphologically and functionally unclear. After 4 months follow-up PET/CT showed successful treatment of sarcoidosis with reduced 1^18^F FDG uptake (SUVmax 4.2) and decreased size of bi-hilar lymph nodes (not shown). During follow-up, Tg steadily increased and an additional ^18^F FDG PET/CT during follow-up revealed positive pulmonary lesions (SUVmax 3.4; **b**) which were iodine-refractory. The patient showed progressive disease during follow-up with local recurrence (SUVmax 19.9), progressive pulmonary metastases (SUVmax 14.5) and hilar lymph node metastases (SUVmax 55.3; **c**) in the ^18^F FDG PET/CT. Tg correspondently increased to 2434 ng/ml. After resection of central lymph node metastases, external radiation therapy of mediastinal lymph nodes and systemic therapy with Lenvatinib (Lenvima® 14 mg/d, Eisai GmbH, 60,528 Frankfurt, Germany) the patient showed partial remission in the PET/CT scan (not shown) and a significant decrease of Tg (12.3 ng/ml)

Physicians were able to differentiate between SA and metastatic thyroid cancer in 10 of 16 patients (63%; 2, 4–6, 8, 10, 11, 14–16). In detail, four patients suffered from active SA (# 2, 4, 6, 16). In these four patients recurrent / persistent TC was excluded. Three patients had recurrent / persistent TC and inactive SA (#5, 14, 15). Two patients were diagnosed with recurrent / persistent TC and active SA simultaneously (#8, 10; Figs. 1 and 2). Last, one patient had inactive SA and was in complete remission of TC (#11). In contrast, in six patients (37%; #1, 3, 7, 9, 12,13) the differentiation between the two entities was not possible despite multiple physical examinations, imaging, laboratory tests and interdisciplinary treatment during regular follow-up visits. In this patient group patients reported an increased psycho-oncological distress score of at least 6/10 points (minimum 6/10; maximum 9/10 points). In comparison, in patients with definite final diagnosis of SA or TC the psycho-oncological distress score ranged slightly lower from 4/10 to 7/10 points.

#### Tumormarker thyroglobulin

In all patients tumour marker Tg was monitored regularly. Six patients presented with negative Tg and Tg recovery in normal range during follow-up (# 3, 5, 6, 11, 14, 15). Five of them had no evidence of thyroid cancer during follow-up. Only one patient (#3) presented with ^18^F FDG positive lymphadenopathy and pulmonary lesions which were unsuccessfully biopsied twice and final diagnosis remained unclear.

Two patients presented with initially negative Tg one with disturbed recovery (#4) and one with Tg antibodies (#1), respectively. Tg antibodies were found in 4/9 patients (#1, 2, 10, 16). In these patients the use of Tg was restricted.

Notably, six patients (#7–10, 12, 13) presented with persistent Tg. Four of them (# 7, 9, 12, and 13) had the confirmation of concomitant active sarcoidosis, and the increased Tg in these patients, usually indicating persistent or recurrent TC, could not be clarified. Two of them (#8, 10) presented with both active SA and TC which were each proven histopathologically.

#### Histopathology and cytology

During follow-up of TC, suspicious lesions were examined histopathologically or cytologically in seven patients. Three of these seven patients (43%; #2, 6, 10) presented with suspicious lesions of either the lung or mediastinal lymph nodes (*N* = 1 lung, *N* = 2 mediastinal lymphadenopathy). In all these patients histopathology clarified the entity. However, four patients (57%, #3, 7, 8, and 9) presented with increased Tg and with suspicious lesions of multiple organ systems involving different lymph node stations and the lung. In these patients only one suspicious lesion was examined confirming SA. Despite increased Tg, indicating recurrent TC, further biopsies separately for the other suspicious lesions were not performed. In patient #8 iodine positive cervical lymph nodes confirmed recurrent TC and explained the increased Tg, while atypical lung resection revealed simultaneous SA (Fig. 1). In the other three patients (# 3, 7, 9) increased Tg remained unclear.

#### Post therapeutic scan of RIT

A total of 34 cycles of ^131^I were administered (cumulative dose 6658 MBq, 180 mCi; 2067–35,510 MBq ^131^I] 200 mCi; 56–960 mCi). In our study, positive ^131^I uptake was seen in the thyroid bed (*N* = 16 after 1st RIT, *N* = 3 after 2nd RIT) corresponding to remnant thyroid tissue. Furthermore, positive cervical uptake in one patient (*N* = 1; #8) was related to a cervical lymph node metastasis. On the contrary, missing iodine uptake of enlarged lymph nodes was seen in one patient with lymph node metastases (#9) and in one with sarcoidosis (#10). Of note, in seven patients (# 1, 2, 8–10, 12, 13; PTC *N* = 4, OFTC *N* = 1, FTC *N* = 2) we have noticed iodine-negative pulmonary lesions in the initial post-therapeutic SPECT/low-dose-CT which were at first clinically classified as iodine-negative metastases. Six patients (# 2, 8–10, 12, 13) were consequently treated with additional cycles of RIT as physicians explained the lack of pulmonary iodine uptake by the sink effect. However, during follow-up two patients (#2, 8, both PTC) had to be downgraded after sarcoidosis was histopathologically confirmed through atypical lung resection. In case of the other five patients (# 1, 9, 10, 12, and 13) histopathological clarification of the lung was not performed. Notably, these patients presented with an increased Tg or increased Tg antibodies (Table 1).

#### ^18^F FDG PET/CT

Twenty-two ^18^F FDG PET/CT scans were performed in eight patients (#2, 3, 5–10) during follow-up. Pulmonary lesions and lymph nodes of either sarcoidosis or TC have shown indistinguishable increased uptake (Fig. 1c, Fig. 2a-c). We furthermore noticed a missing uptake in small lung nodules independently of the pathogenesis. Retrospectively, ^18^F FDG positive mediastinal, bilateral hilar lymphadenopathy and pulmonary lesions have been misclassified as metastatic thyroid cancer although these findings can arise from many other causes including sarcoidosis. The clinical anamnesis of thyroid cancer and the missing information of co-existent sarcoidosis as secondary referral diagnosis have retrospectively influenced reporting physicians. However, in three patients (#3, 9, 10) ^18^F FDG PET/CT proved to be helpful in assessing the clinical course and treatment response of both sarcoidosis and TC (Fig. 2).

#### Clinical follow-up

All patients were followed up regularly. In patients who refused histopathological clarification of suspicious lesions final diagnosis had to be established during long-term follow-up.

In four patients (#4, 5, 15, 16) physicians were able to assign pulmonary lesions or lymphadenopathy to the final diagnosis of SA (*N* = 2) or metastatic thyroid cancer (*N* = 2) during long-term follow-up. In patients # 5, 15 suspicious lesions were iodine positive and Tg levels corresponded to the clinical course after repeated RIT. Patients # 4, 16 presented with suspicious lesions of the lung or lymph nodes and Tg was under the detection limit. During long term follow-up no significant changes in these lesions were detected. Pulmonary work up was not specific but could not exclude recurrent or chronic SA. Finally, lesions were assigned to SA.

In three patients (#1, 12, 13) a final diagnosis of either SA or TC could not be established during long-term follow-up. All three patients presented with iodine-negative pulmonary lesions. Pulmonary work up was not specific for active SA. In detail, in patient #1 (PTC) pulmonary lesions were progressive during long-term follow-up. However, Tg was not measurable due to increased Tg antibodies. Furthermore, the clinical course was not clearly indicative for a progressive active sarcoidosis (inconspicuous pulmonary function testing in body plethysmography, no significant findings in the provocation test with methacholine, no reduced diffusion capacity for carbon monoxide, ACE within the normal range, increased interleukin-2-receptor only). Patients #12 presented with increasing Tg and with iodine and ^18^F FDG negative pulmonary lesions. In patient #13 Tg was above the detection level and ^18^F FDG PET/CT revealed increased uptake in the cervical, hilar and mediastinal lymph nodes while pulmonary lesions were negative. Finally, physicians were not able to differ between SA and TC in these patients without histopathological clarification.

#### Time point of diagnosing SA

Initial diagnosis of sarcoidosis was established in 6/16 patients (# 5–7, 11, 12, 16) before the diagnosis of TC and in 2/16 patients simultaneously (# 13, 15). Thus, in these patients treating physicians of TC were aware of the diagnosis of sarcoidosis. In this subgroup, physicians were able to differentiate between recurrent or persistent TC and SA in 63% of patients (# 5, 6, 11, 15, 16).

In half of our patients sarcoidosis was diagnosed during the follow-up of TC (# 1–4, 8–10, and 14). In 88% (7/8) patients of the latter subgroup differentiation of sarcoidosis and TC was highly challenging. Two patients were falsely upgraded to cM1 of the lungs and had to be downgraded during follow-up (#2, 8). In two patients final diagnosis could only be established during long-term follow-up and after repeated examinations (#4, 10). However, finally the differentiation between sarcoidosis and metastatic TC was possible in five patients (63%; #2, 4, 8, 10, 14).

## Discussion

Clinical and radiographic manifestation of sarcoidosis and metastatic TC can mimic each other, making the differential diagnosis between the two challenging in certain cases. Lymph node metastases, distant pulmonary and bone metastases are the most common sites of TC metastases while brain and liver metastases are seen far less often. Sarcoidosis is a systemic disorder affecting various organs which is characterized by the development of granulomas. The lungs are most commonly involved in 90% of patients [19]. Furthermore, hilar and mediastinal lymphadenopathy is also observed regularly. Hereby, more than 20% of patients have peripheral lymphadenopathy which can also involve the cervical lymph nodes [3].

Up to now, the co-existence of TC and sarcoidosis has been described only in individual case reports [9–17]. They stated that clinicians should be aware of the co-existence of SA and TC to avoid mismanagement and recommended a thorough work up including biopsy and histopathology examinations separately for each suspicious lesion. Thus, this is the first study to describe the difficulties in diagnosing and management of co-existing TC and sarcoidosis in a larger patient group of 16 patients at a single centre.

Sarcoidosis and malignancy have been associated in some case series including patients diagnosed with cancer directly before or after the diagnosis of sarcoidosis. However, this relationship remains controversial and may be incidental [20–25]. Hypothesis of a possible association and a possible increased risk of cancer include the chronic inflammation, the immune system dysregulation and immunosuppressive medication of sarcoidosis. In our study, sarcoidosis was known before or at time point of TC diagnosis in only half of our patients. Thus, we suggest that the coexistence of TC and sarcoidosis at least in the other half of our patient group is likely to be incidental. Furthermore, there might be a possible relationship between sarcoidosis and autoimmune disorders, especially Hashimoto’s thyroiditis which has been discussed in a small number of studies [26, 27]. In this setting, in other studies including patients with SA and autoimmune thyroiditis, a remarkably high incidence of positive autoantibodies against thyroid peroxidase and thyroglobulin were found [27–31]. In our study, we found Tg antibodies in a relevant number of patients. Normally, Tg antibodies can be present in up to 30% of patients with TC [32–34]. If patients with concomitant SA are affected more often needs to be clarified in further studies. However, in our patients with increased Tg antibodies the use of the tumour marker Tg was of limited value aggravating the differentiation between SA and thyroid cancer. Measurement of Tg by mass spectrometry assays (LC-MS/MS) has been introduced as a solution for accurate Tg quantitation in the presence of Tg antibodies and might be helpful in this subgroup. Of note, in our patients presenting with negative Tg without disturbed recovery and in the absence of Tg antibodies recurrent thyroid cancer could be excluded with high probability.

Furthermore, we observed that ^18^F FDG PET/CT was not helpful to distinguish between both entities, especially if they both coexisted. In our study ^18^F FDG PET/CT showed positive and negative uptake in both entities. ^18^F FDG accumulates in inflammatory and tumour cells and therefore allows both the visualization of inflammatory and tumour activity [35, 36]. Additionally, rare sarcoid-like reactions 6–86 months after the treatment of malignancies are also described in ^18^F FDG PET/CT [37] making a differential diagnosis even more challenging. However, in three patients ^18^F FDG PET/CT was helpful in monitoring the course of both diseases and treatment response.

Positive iodine uptake in the post-therapeutic ^131^I WBS (and SPECT/low-dose-CT) was indicative of remnant thyroid tissue or metastatic TC. In contrast, in our selected patient group lack of iodine uptake was unspecific and could be either lesions of SA or metastatic TC. Lung nodules in the post therapeutic iodine SPECT/low-dose-CT scan have been misinterpreted as iodine-negative distant pulmonary metastases (cM1). In fact, missing iodine-uptake in pulmonary metastases at 1st RIT may be potentially attributed to the well-known sink effect or interpreted as iodine refractory metastasis. Misclassification especially occurred when physicians focused on TC during follow-up and possible differential diagnosis were not directly taken into consideration. However, upgrading to cM1 has massive consequences with a more aggressive treatment regime including TSH suppression, further staging examination, additional RIT or even redifferentiation therapy.

Histopathology and cytology distinguished reliably between SA and thyroid cancer in patients with one suspicious lesion. However, if Tg was increased and patients presented with more than one suspicious lesion it was not sufficient to examine only one lesion. Therefore, we believe that each lesion should be histopathological examined or biopsied separately. In case of biopsies only cytological examinations were performed in our clinic. However, several studies have reported that detection of Tg in fine-needle aspiration (FNA) biopsy washout fluid from lymph nodes identifies recurrences/metastases of differentiated TC [38, 39]. The combination of fine-needle lymph node aspiration for cytology and for thyroglobulin measurement further increases diagnostic accuracy [40–42]. Thus, in future studies ultrasound or CT guided FNA for thyroglobulin measurement and for cytology should be examined in this selected patient group.

In patients who refused biopsy the long clinical follow-up was helpful in a relevant number of patients. However, if the differentiation between SA and TC is not possible patients reported an increased psycho-oncological distress score of at least 6/10 points. A score of 5 is internationally recommended as an indicator that a patient is distressed and needs support [18, 43]. Therefore, an interdisciplinary treatment team consisting of nuclear medicine physicians, endocrinologists, oncologists, pneumologists, radiologists, and clinical psychologists is favourable in this rare patient group.

### Limitations

A limitation arises from the retrospective study design. Due to the retrospective design we might have missed TC patients with co-existent sarcoidosis if not documented in the medical record. Furthermore, Tg antibodies were not available in all patients. If multiple lesions existed histopathological biopsy was not obtained separately for each lesion.

## Conclusion

Sarcoidosis can mimic metastatic disease of TC to lymph nodes and lung and should be considered in the differential diagnosis of radioiodine negative lymph node or pulmonary lesions on a ^131^I posttherapy scan especially when these findings do not correlate with Tg measurements that can however be also hampered by the presence of Tg antibodies. ^18^F FDG PET/CT proved to be helpful to monitor treatment success but not in the differentiation between TC and sarcoidosis. Histopathology distinguished reliably between SA and thyroid cancer. If multiple organs are involved each lesion should be biopsied separately. In half of our patients who refused histopathological clarification a closely monitoring during follow-up was helpful to distinguish between both entities clinically.

## Data Availability

The datasets generated during and/or analyzed during the current study are available from the corresponding author or first author on reasonable request.

## Abbreviations

Bq: Becquerel
CI: Curie
^18^F FDG: 2-deoxy-2-^18^F-fluoro-D-glucose
FTC: Follicular thyroid cancer
^131^I: Radioiodine
LAE: Lymphadenectomy
M: Metastasis
N: Nodus
NCCN: National Comprehensive Cancer Network
PTC: Papillary thyroid cancer
R: Resection
rhTSH: recombinant human thyrotropin alfa
RIT: Radioactive iodine therapy
SA: Sarcoidosis
TC: Thyroid cancer
Tg: Thyreoida globuline
TSH: Thyreoidea stimulating hormone
WBS: Planar whole-body scans
